# A Preliminary Study about the Role of Reactive Oxygen Species and Inflammatory Process after COVID-19 Vaccination and COVID-19 Disease

**DOI:** 10.3390/clinpract12040063

**Published:** 2022-08-04

**Authors:** Evgenia Lymperaki, Konstantina Kazeli, Ioannis Tsamesidis, Polykseni Nikza, Irini Poimenidou, Eleni Vagdatli

**Affiliations:** 1 Department of Biomedical Sciences, International Hellenic University, 57400 Thessaloniki, Greece; 2 School of Physics, Faculty of Sciences, Aristotle University of Thessaloniki, 54124 Thessaloniki, Greece; 3 Department of Prosthodontics, School of Dentistry, Faculty of Health Sciences, Aristotle University of Thessaloniki, 54124 Thessaloniki, Greece; 4 Nea Michaniona Health Care Centre, 57004 Nea Michaniona, Greece

**Keywords:** COVID-19, ROS, mRNA vaccines, antibodies

## Abstract

During the last couple of critical years, worldwide, there have been more than 550 million confirmed cases of COVID-19, including more than 6 million deaths (reported by the WHO); with respect to these cases, several vaccines, mainly mRNA vaccines, seem to prevent and protect from SARS-CoV-2 infection. We hypothesize that oxidative stress is one of the key factors playing an important role in both the generation and development of various kinds of disease, as well as antibody generation, as many biological paths can generate reactive oxygen species (ROS), and cellular activities can be modulated when ROS/antioxidant balance is interrupted. A pilot study was conducted in two stages during the COVID-19 pandemic in 2021 involving 222 participants between the ages of 26 and 66 years. ROS levels were measured before an after vaccination in the blood samples of 20 individuals who were vaccinated with two doses of mRNA vaccine, and an increase in ROS levels was observed after the first dose, with no modifications observed until the day before the second vaccination dose. A statistically significant difference (*p* < 0.001) was observed between time points 3 and 4 (before and after second dose), when participants were vaccinated for the second time, and ROS levels decreased from 21,758 to 17,580 a.u. In the second stage, blood was collected from 28 participants 45 days after COVID-19 infection (Group A), from 131 participants 45 days after receiving two doses of mRNA vaccine against COVID-19 (Group B), and from 13 healthy individuals as a control group (Group C). Additionally, antibodies levels were measured in all groups to investigate a possible correlation with ROS levels. A strong negative correlation was found between free radicals and disease antibodies in Group A (r = −0.45, *p* = 0.001), especially in the male subgroup (r = −0.88, *p* = 0.001), as well as in the female subgroup (r = −0.24, *p* < 0.001). Furthermore, no significant correlation (only a negative trend) was found with antibodies derived from vaccination in Group B (r = −0.01), and a negative trend was observed in the female subgroup, whereas a positive trend was observed in the male subgroup.

## 1. Introduction

SARS-CoV-2, like other coronaviruses, is an enveloped virus with a genome length of approximately 29.8 kb and can cause diseases ranging from the common cold to severe and fatal illnesses in humans [1,2,3,4]. Structurally, the virus consists of a spike glycoprotein, and the envelope’s membrane is made of M and E proteins and a 5–7 nm length of S protein. The S1 subunit interacts with angiotensin-converting enzyme 2 (ACE2) and the S2 subunit of S protein, in addition to playing an important role in the viral entry process. It is also highly immunogenic because it acts as a target for antibody-mediated neutralization; these functions make the S1 subunit a suitable candidate for the design of vaccines and therapies [5,6,7,8,9,10,11,12].

When the S protein binds to the ACE2, which plays the role of membrane receptor, it stimulates the production of excessive reactive oxygen species (ROS) in an NADPH (NOX2)-dependent manner [13]. This production induces ROS-dependent cellular signaling, including activation of the NF-kB (nuclear factor kappa-light-chain-enhancer of activated B cells) pathway, which amplifies inflammation through the expression of multiple genes, TNF-α (tumor necrosis factor), interleukins (IL, IL2, IL6, IL7, IL8, IL9, and IL10), chemokines (CXCL10, CCL2, IP10, and MCP1), and colony-stimulating factors (G-CSF and GM-CSF), all of which induce endothelial injury and/or dysfunction, as well as vascular inflammation [14,15]. NOX2-mediated excessive ROS production in endothelial cells generated by SARS-CoV-2 infection combined with a dysregulation and/or extremely exaggerated immune imbalance (so-called cytokine response or cytokine storm) might be among the most important mechanisms of endothelial cell injury during COVID-19 [16,17]. This phenomenon successively aggravates disease progression, activates immune and endothelial cells and platelets, as well as vigorous endothelial inflammatory reactions, which may cause oxidative stress (OS), leading to inflammatory injury and vascular thrombosis and possibly driving ARDS (acute respiratory distress syndrome) or multi-organ failure, pulmonary edema, and, ultimately, mortality [18,19,20,21]. Oxidative stress (OS) can be induced by different viruses and infections, such as influenza [22], human respiratory syncytial virus [23], and rhinoviruses [24], which employ diverse mechanisms in the human body/organism and may profoundly impact COVID-19 pathogenesis. Additionally, numerous data have confirmed that the development of OS can lead to cytokine production, cell death, inflammation, and other pathophysiological processes, with which most respiratory-based viral COVID-19 infections can be in strong harmony [25,26]. Following these pathophysiological processes, an excessive amount of reactive oxygen species (ROS) is produced in various tissues [27], and this overproduction of ROS and the mechanism of antioxidant deprivation can lead to serious, life-threating results of the subsequent virus-associated disease, i.e., COVID-19 [28,29]. Therefore, OS plays a leading pathogenic role in viral infections, such as COVID-19, and consequently weakens the antioxidant system [30]. More precisely, ROS can cause inflammation, biological tissue damage, and cell apoptosis [31,32]. Some studies have shown that high concentrations of ROS cause damage not only in “healthy” tissue and cells but also in infected cells and even the virus itself by using retroviruses’ DNA and RNA [33].

mRNA= and adenovirus-based vaccine platforms have been approved now for human use [34,35]. A considerable amount of previous research has shown that type I interferons (IFN) [17] transiently increased after vaccination against viruses, such as influenza [36,37], and can activate an adaptive immune response and influence neutralizing antibody production [38]. However, the direct effect of vaccination upon the formation of intracellular OS has not been sufficiently studied to date. According to the literature, the four COVID-19 vaccines are based mainly on mRNA and DNA technologies, and sufficient protection from viral infection is achieved by the viral neutralizing antibody principle of immune vaccination by inducing mRNA cells that generate spike proteins, triggering antibody production [39].

In mRNA-based vaccines, the mRNA molecules are injected directly into the host cell and translated into the target protein in the cytoplasm. The overall design of mRNA vaccines contains an open reading frame (ORF) with a 3′poly-adenylated tail that can induce both cellular and humoral immune responses [40,41]. Some types of vaccines, such as the Moderna and Pfizer vaccines, are designed to function as nanocarriers, inducing B- and T-cell immune response [34,42,43] by embedded lipid nanoparticles (LNPs) and encoding the membrane-anchored, full-length S protein (BNT162b2) and secreted receptor-binding-domain (BNT162b1) of SARS-CoV-2 [11,43,44].

Taking into consideration that immune cells induce cytokines and ROS production, their correlation was investigated. The aim of this study was to identify whether ROS could be used as a potential biomarker for early indication of the immune response after infection and vaccination.

## 2. Materials and Methods

### 2.1. Study Design

A pilot study (Table 1) was conducted in the General Hospital of Thessaloniki, Greece, from April 2021 to June 2021 during the COVID-19 pandemic. The participants (*n* = 222) were comprised of 103 males and 119 females aged 26–66 years. The study was divided into two stages. In the first stage (1st study), 20 individuals (9 males and 11 females aged 28–59 years) vaccinated with two doses of mRNA (10 with Pfizer and 10 with Moderna) vaccines participated in our study. Blood collection was performed 1day before vaccination, 3 days after the first dose, 1day before the second dose, and 3 days after the second dose. Once the data were analyzed, in the second stage (second study), blood was collected from 161 of participants (79 males and 82 females aged 25–66 years) (Group B) 45 days after vaccination with the 2nd dose of them RNA vaccine against COVID-19, according to other studies [41]. Moreover, in our study, we included the blood specimens of 28 infected participants (with mild severity) unvaccinated against COVID-19(10 male, 18 female) (Group A) and 13 apparently healthy individuals who had never been infected or vaccinated (5 male, 8 female) as controls (Group C). All participants were healthy and provided written informed consent before the study and were asked to fill out a short questionnaire about sociodemographic characteristics, such as age and sex.

### 2.2. Ethical Statement

All participants (all adults) provided written informed consent before entering the study. The Research Quality Control Committee of the General Hospital of Thessaloniki2042021 approved the study. The study was conducted in accordance with Good Clinical Practice guidelines and the Declaration of Helsinki. The confidentiality of participants was wholly preserved.

### 2.3. Analysis of Antibodies

All samples were tested for SARS-CoV-2 IgG antibodies against the spike protein by chemiluminescent microparticle immunoassay (CMIA)on an Alinity ABBOT automated molecular diagnostic analyzer. The assay was performed according to manufacturer’s instructions. All antibodies were measured in U/mL, and a positive result was considered for values ≥50 U/mL. Following blood collection, serum of all samples was separated and stored at −80 °C until assays were performed.

### 2.4. Analysis of ROS Levels

Reactive oxygen species were analyzed using a cell-permeable, ROS-sensitive 2′,7′-dichlorodihydrofluorescein diacetate probe (H2DCFDA) fluorescing at 520 nm (λ_ex_ 480 nm) upon oxidation. An H_2_DCFDA probe (0.5 mM stock solution in DMSO) (incubated for 30 min) was incubated in the human plasma containing the plasma membrane vesicles (PMVs) responsible for the ROS presence, as previously described [45]. ROS levels were analyzed at all tested time points. All plasma samples were preserved at −80 °C. Measurement of the fluorescence of the desired suspensions was monitored in 96-well black microplates using a Tecan fluorometer. All data were evaluated at mM of ROS in accordance with the standard curve of H_2_O_2_in a concentration range of 0–3mM.

### 2.5. Statistical Analysis

SPSS tool version 22.0 was used to compare and correlate the ROS levels of the different groups (stage 1, the participants at different time points of investigations) and (stage 2, Groups A to C) with the antibody measurements (when available). Descriptive statistics were performed, presented as means ± standard deviations. Additionally, inferential statistical analysis (*t*-test) was used to investigate the possible differences between the groups. Pearson’s chi-square test or chi-square test of association we reused to determine whether there was a relationship between the categorized data, whereas Fisher’s exact test was used when expected variables accounted for 2% of the total number of variables. In all statistical analyses, the level of significance (*p*-value) was set at α = 0.05.

## 3. Results

### 3.1. First Study

**Figure 1:**
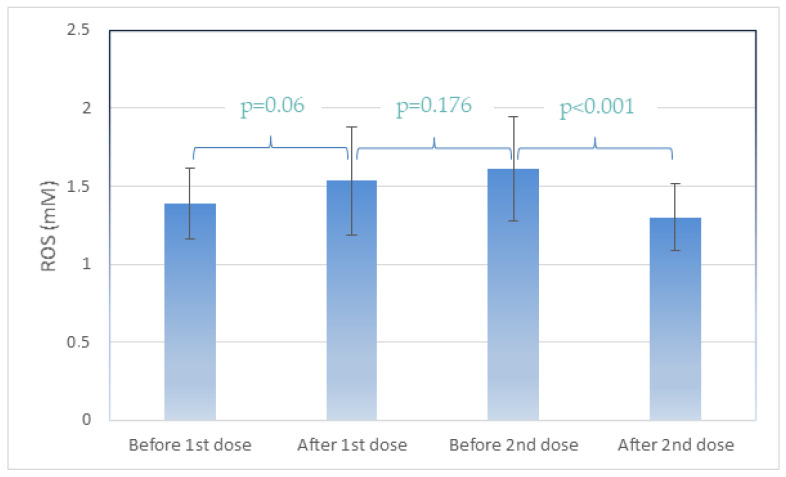
Average ROS (Reactive Oxygen Species) levels at the four timepoints. Data are represented as mean (±SD).

Table 2 presents the antibody levels in U/mL and the reactive oxygen species (ROS) levels detected in human plasma containing the plasma membrane vesicles (PMVs) responsible for the ROS presence, as previously described [46]. Participant follow-up from time 1 (before 1st dose), time 2 (after first dose), time 3 (before second dose), and time 4 (after second dose) revealed significant differences. An increase in ROS levels was observed after the first dose between time points 1 and 2 (*p* = 0.06). ROS levels were unchanged from the first dose until the day before the second vaccination dose (times point 2 and 3, *p* = 0.176). A statistically significant difference (*p* < 0.001) was observed between time points 3 and 4, when participants were vaccinated for a second time, with a decrease in ROS levels (from 1.612 to 1.302 mM *p* < 0.001). (Figure 1). Antibody levels were extremely low (<50 U/mL) before and after the first vaccination dose, whereas before the second dose, a considerable increase was observed (5000 to 13,000 U/mL), in addition to an increase after the second dose.

### 3.2. Second Study

After we evaluated the ROS levels for each time point after two vaccination doses, we further analyzed more participants after the second dose of mRNA vaccine, appearing as a crucial time point for the ROS levels, and we additionally measured their antibody levels to investigate a possible correlation. Table 3 presents the ROS and antibody levels of participants infected with COVID-19 after 45 days of recovery (Group A); vaccinated participants 45 days after receiving the second dose of mRNA vaccine (Group B); and non-infected, non-vaccinated participants (Group C) as a control. In detail, a strong negative correlation was found between free radicals and disease antibodies in group A (r = −0.55, *p* = 0.001), especially in the male subgroup (r = −0.88, *p* = 0.001) as well as the female subgroup (r = −0.23, *p* = 0.001), whereas no significant correlation was found with antibodies derived from vaccination in group B (r = 0.01, *p* < 0.001). Only a positive trend was observed in the vaccinated group, especially in the male subgroup, whereas a negative trend was observed in the female subgroup. Finally, no statistically significant correlation with a negative trend was found between ROS levels and age (Group B: r = −0.0012; all participants: r = −0.032), except Group A (r = −0.439). Concerning the correlation between antibodies and age, no statistically significant correlation was found (Group A: r = 0.087; all participants: r = −0.0209), except Group B (r = −0.32), also showing a negative trend.

## 4. Discussion

Novel vaccine technologies in the fight against COVID-19 disease lead to innate immunity responses, the mechanisms of which have not yet been fully characterized. Recent studies have shown that vaccinated patients, mostly with two mRNA vaccine doses, were admitted to hospital with COVID-19 with significantly lower disease severity than unvaccinated patients. Consequently, mRNA vaccines considered to be highly effective against COVID-19, preventing and reducing hospital admissions and deaths due to COVID-19 in adults older than 60 years of age [47,48].

In our study, we attempted to understand the effect of mRNA vaccines on ROS response and production. It is of a critical importance to understand the role of OS and its correlation with immune response after vaccination and infection time because of the global vaccination campaign.

All participants were selected based on their health status, excluding those with drug and supplement intake. It was suggested to participants not to change their dietary and daily habits (sleeping hours, smoking, routine, etc.) during the research study to ensure that ROS levels were as unaffected by these external parameters as possible and only vaccination dosage correlation was monitored. The healthy controls had not been vaccinated and had never been infected with COVID-19. Those who did not follow these suggestions became ill in the process were excluded from the study. Our preliminary data after the first stage of analysis (ROS level measurement in a small mRNA-vaccinated group) support the hypothesis that ROS levels are affected by vaccination and may result in a proportional response to antibody production, which prompted us to further investigate the relationship between ROS and antibody production (second stage). Our study was driven by the question of whether ROS could be used as a biomarker of efficient antibody development after vaccination. The first dose of mRNA vaccination resulted in an increase in ROS levels, which remained high until before the second dose, although antibody levels were low after the first dose and increase before the second dose. After the second dose ROS levels seemed to be lower and stabilized, and antibody levels also stabilized. These data are in agreement with other studies concerning antibody levels. Cristina Bergamaschi et al. characterized cytokine and chemokine responses to the Pfizer mRNA vaccine after the first and second dose in antigen-naive and COVID-19-infected individuals and identified increases in IL-15, IFN-γ, and IP-10/CXCL10 after the first vaccination dose, indicating antibody responses to spike-RBD and trimeric spike on the day of vaccination, which was not followed by a further increase in antibody responses upon the second vaccination dose, remained significantly higher than those in the SARS-CoV-2-naive vaccine recipients. This result is in total agreement with the ROS response observed in our preliminary data.

A few studies support the importance of the examination of different parameters after within two days following vaccination because any early response to vaccination (“vaccine signatures”) is important with respect to predicting immunogenicity and can be used to optimizing the efficacy of vaccine strategies. Therefore, we examined ROS production two days after vaccination, and our findings led to the assumption that ROS could possibly be used as an early biomarker to predict vaccine response. The same correlation was found between IL-15 and IFN-g and anti-spike antibody responses in a study by Cristina Bergamaschi et al. [49], in which, by measuring biomarkers within 2 days before and after vaccination and within 1 month after a booster vaccine dose, a correlation was found to be of a considerable importance in order to draw safe conclusions with respect to optimization of vaccine development for public health.

Thus, in the second stage of our study, we investigated ROS 45 days after the second dose, as well as after COVID-19 infection, for all individuals and study groups and found that infection promotes less immune response than a second dose of vaccination, although ROS production was at the same level. These findings are in agreement with results of other studies, suggesting that humoral immune responses are promoted following a second vaccination dose and are more intense compared to those observed in COVID-19 patients with severe disease [41,50].

Additionally, several studies have revealed an age, gender and, seronegativity dependence of antibody response against COVID-19. Additional doses of vaccination are recommended for immunocompromised and elderly populations following a combined scheme of mRNA vaccines [51]. In our study, we confirmed a gender-dependent correlation antibodies with ROS production, both in post-vaccination and COVID-infected individuals. A study by E. Terpos et al. study on the first dose of the Pfizer vaccine showed a triggered robust immune response for 50 days in COVID-19-naive recipients, which was also age- and gender-dependent [10,41], as well as a stronger induction in female versus male vaccine recipients of IFN-g, IL-15, IL-6, and IP-10/CXCL10 upon receipt of a second vaccination dose. The authors found that mRNA vaccination triggers a significant mobilization of adaptive immune responses 22 day after the first vaccine dose, which after the plateaus at higher values after the second dose not only in COVID-19 patients but even in COVID-19-infected patients with severe disease. In a study on health workers and octogenarians after vaccination with an mRNA vaccine, Evangelos Terpos et al. [52] showed that antibody response against SARS-CoV-2 was age-dependent and gender-dependent. They concluded that mRNA vaccines produce high levels of anti-SARS-CoV-2, as well as anti-RBD IgG and NAb titers after the first dose, which is similarly more robust in younger patients and in female octogenarians.

Table 3 shows that ROS production seems to decrease faster after a 45-day period after the disease rather than after vaccination. Experimental results indicate different behavior between men and women after vaccination. Our results show that after vaccination, women return to normal levels of ROS faster than men, and although they have more antibodies than men, ROS levels are also lower. After disease, in both genders, the preliminary data indicate a generally lower level of antibodies and ROS production, whereas the more antibodies produced, the faster the organism returns to normal ROS levels. Nonetheless, more data are needed in order to draw more solid conclusions because free radicals are normally produced during the production of antibodies, andbodily mechanisms attempt to neutralize them. During disease, the production of antibodies is controlled, and thus, the body can reduce ROS levels faster than it can after vaccination. There is probably a faster neutralization mechanism in the female population.

## 5. Conclusions

Our data suggest that attenuating ROS levels in combination with antibodies could lead to preliminary conclusions about interactions with immune cells. An increase in ROS levels was observed after the first dose, and this increase was not attenuated until the day before the second vaccination dose, indicating the crucial role of ROS in the process of immunization. A strong negative correlation was found between ROS and disease antibodies for both sexes, highlighting the importance of natural immunization in equilibrating ROS levels.

## Figures and Tables

**Table 1:** Description of pilot study.

	1st Study	2nd Study		
	Vaccinated (2 Doses)	Infected and Unvaccinated Group A	Vaccinated (2 Doses) Group Β	Healthy Control Group C
SEX				
Male	9	10	79	5
Female	11	18	82	8
Age				
25–40	12	8	51	4
40–66	8	20	110	9
Total participants	20	28	161	13

**Table 2:** Reactive oxygen species (ROS) levels (mM) and antibody levels (U/mL) of selected participants (*n* = 20). Time point 1: participants before the first dose of mRNA vaccine against COVID-19; time point 2: participants after the first dose; time point 3: participants before the second dose; and time point 4: participants after the second dose. Data are the averages ± SDs of the four measurements. Significant differences between time points 3 (before second dose) and 4 (after second dose) are represented as *p* < 0.01.

	Before First Dose		After First Dose		Before Second Dose		After Second Dose	
	Time Point 1		Time Point 2		Time Point 3		Time Point 4	
Participant	Reactive Oxygen Species (ROS) [mM]	Antibodies	Reactive Oxygen Species (ROS) [mM]	Antibodies	Reactive Oxygen Species (ROS) [mM]	Antibodies	Reactive Oxygen Species (ROS) [mM]	Antibodies
		U/mL		U/mL		U/mL		U/mL
ID1	1.634	40	2.146	50	2.127	5660	1.714	6330
ID2	1.137	6.3	1.521	13	1.502	13470	1.236	13,250
ID3	1.489	4.6	0.603	4.5	1.110	12780	1.285	13,250
ID4	1.089	3.8	1.455	4.3	1.436	9500	1.547	9400
ID5	1.588	7.5	1.714	8.5	2.081	6780	1.314	6800
ID6	1.072	6.5	1.252	6.2	1.269	8200	0.847	8500
ID7	1.318	2.8	1.787	3.2	1.870	5800	1.299	5300
ID8	1.574	3.7	2.112	2.7	2.272	4500	1.53	5400
ID9	1.354	37	1.471	19	1.498	7150	1.285	7200
ID10	1.235	15	1.293	14.5	1.356	12000	1.225	11,980
ID11	1.872	1.8	1.890	2.7	1.990	5400	1.446	5600
ID12	1.428	2.8	1.618	2.9	1.656	6800	1.300	6700
ID13	1.307	0.6	1.291	0.6	1.302	12700	1.056	11,900
ID14	1.137	0.7	1.317	0.9	1.373	11150	1.147	11,650
ID15	1.737	16	1.861	10.3	1.821	6750	1.490	6700
ID16	1.291	2.8	1.360	14	1.389	8500	1.228	8400
ID17	1.152	6.3	1.302	10.5	1.323	10.900	1.005	11,110
ID18	1.433	8.5	1.656	9.5	1.589	7200	1.384	7300
ID19	1.325	9.5	1.426	9.4	1.375	11.500	1.080	11,150
ID20	1.574	28	1.590	17.8	1.887	7200	1.618	7150
Average	1.387	23.7	1.533	24	1.611	8697	1.302	8735
SD	0.224		0.346		0.331		0.216	

**Table 3:** Reactive oxygen species (ROS) levels (a.u) and antibodies (U/mL) of selected participants (*n* = 202). Group B, participants 45 days after infection with COVID-19; Group A, vaccinated participants 45 days after receiving the vaccine dose; Group C, non-infected, non-vaccinated controls. Significant differences are represented as *p* < 0.001.

		ROS (mM)	Antibodies (U/mL)	r-Value	*p*-Value
			Mean		
Group A (*n* = 28)		1.213	6584	−0.55	0.001
Male	10	1.373	10,642	−0.88	0.001
Female	18	1.434	5076	−0.23	0.001
Group B (*n* = 161)		1.243	12,179	0.01	<0.001
Male	79	1.419	9668	0.1	<0.001
Female	82	1.194	13,178	−0.00044	0.01
Group C (*n* = 13)		0.885	6.26	−0.24	<0.0001

## Data Availability

Not applicable.
